# Nature and Nurture: Genotype-Dependent Differential Responses of Root Architecture to Agar and Soil Environments

**DOI:** 10.3390/genes12071028

**Published:** 2021-07-01

**Authors:** Merijn Kerstens, Vera Hesen, Kavya Yalamanchili, Andrea Bimbo, Stephen Grigg, Davy Opdenacker, Tom Beeckman, Renze Heidstra, Viola Willemsen

**Affiliations:** 1 Cluster of Plant Developmental Biology Laboratory of Molecular Biology, Wageningen University & Research, Droevendaalsesteeg 1, 6708 PB Wageningen, The Netherlands; merijn.kerstens@wur.nl (M.K.); vera.hesen@wur.nl (V.H.); kavya.yalamanchili@wur.nl (K.Y.); andrea.bimbo@wur.nl (A.B.); stephen.grigg@wur.nl (S.G.); renze.heidstra@wur.nl (R.H.); 2 Department of Terrestrial Ecology, Netherlands Institute of Ecology, 6700 AB Wageningen, The Netherlands; 3 Department of Plant Biotechnology and Bioinformatics, Ghent University, 9052 Ghent, Belgium; davy.opdenacker@psb.vib-ugent.be (D.O.); tom.beeckman@psb.vib-ugent.be (T.B.); 4 VIB Center for Plant Systems Biology, 9052 Ghent, Belgium

**Keywords:** root system architecture, *Arabidopsis thaliana*, rhizotron, PLETHORA, geometric morphometrics

## Abstract

Root development is crucial for plant growth and therefore a key factor in plant performance and food production. *Arabidopsis thaliana* is the most commonly used system to study root system architecture (RSA). Growing plants on agar-based media has always been routine practice, but this approach poorly reflects the natural situation, which fact in recent years has led to a dramatic shift toward studying RSA in soil. Here, we directly compare RSA responses to agar-based medium (plates) and potting soil (rhizotrons) for a set of redundant loss-of-function *plethora* (*plt*) CRISPR mutants with variable degrees of secondary root defects. We demonstrate that *plt3plt7* and *plt3plt5plt7* plants, which produce only a handful of emerged secondary roots, can be distinguished from other genotypes based on both RSA shape and individual traits on plates and rhizotrons. However, in rhizotrons the secondary root density and the total contribution of the side root system to the RSA is increased in these two mutants, effectively rendering their phenotypes less distinct compared to WT. On the other hand, *plt3*, *plt3plt5*, and *plt5plt7* mutants showed an opposite effect by having reduced secondary root density in rhizotrons. This leads us to believe that plate versus rhizotron responses are genotype dependent, and these differential responses were also observed in unrelated mutants *short-root* and *scarecrow*. Our study demonstrates that the type of growth system affects the RSA differently across genotypes, hence the optimal choice of growth conditions to analyze RSA phenotype is not predetermined.

## 1. Introduction

Plant roots are responsible for nutrient and water uptake and are thus critical components of overall plant productivity [1]. Understanding the fundamental mechanisms regulating root system architecture (RSA) is important for future crop improvement [2]. *Arabidopsis thaliana* (Arabidopsis) is the most widely used plant model for studying fundamental processes in plant biology. The relative simplicity of the Arabidopsis root system makes it an ideal candidate to identify new players in RSA and to study the impact of endogenous traits and/or exogenous factors on root development. The Arabidopsis root system consists of two main classes of roots that together constitute the RSA, the primary root and the side roots. Side roots can be further classified into secondary roots that branch off the primary root directly and higher-order roots. Depending on the molecular pathway and tissue of origin, secondary roots can be either lateral roots (LRs) or lateral adventitious roots. If side roots are derived from non-root tissue, they are called adventitious roots [3].

To study the RSA in Arabidopsis, in vitro culture is the most common method [4]. Seedlings are grown vertically in Petri dishes with agar-based medium (plates), causing roots to develop along the surface of a gelatinous medium in a 2D space suitable for image-based analyses and easy harvesting. Despite these advantages, in vitro methods differ vastly from the natural situation Arabidopsis normally grows in. On plates, roots are exposed to light instead of below-ground darkness, which can influence their development [5]. Furthermore, the agar medium is smooth, homogeneous, and sterile, in stark contrast to the heterogeneity of particles, water, nutrients, and microorganisms found in soil. These factors also act as signals that shape 3D root growth to optimize uptake. Additionally, the confined atmosphere in plates limits gas exchanges and metabolism within plants, and the size of the plates limits the studies to young plants (generally 2 to 3 weeks). These differences of in vitro versus the natural environment have led to the tendency that more and more RSA studies are performed in a soil environment. However, studying RSA in soil is challenging because imaging it is more complex. In the last few years, soil-based 2D or 3D methods, such as rhizotrons [6,7] or X-ray tomography [8], have been adapted to Arabidopsis. A transparent solid medium was developed to facilitate the observations [9,10]. Major drawbacks of the existing soil-like methods are their reduced flexibility, the fact that the roots are less accessible for imaging, and the fact that most of the described sophisticated methods are expensive and not readily available [10]. Although the tendency is to switch to a more “natural” environment for analyzing the RSA, the in vitro plate and the soil-based methods have not been directly compared in root architecture mutants. This comparison is important, because it can show whether previously described plate phenotypes should be revisited in a soil-based study and whether future studies should be performed preferably on soil or in traditional vitro culture. 

In Arabidopsis, the PLETHORA (PLT) proteins have been shown to be crucial for root development, controlling both specification and maintenance of root meristems as well as priming and outgrowth of LRs [11,12,13]. PLT3, PLT5, and PLT7 are regulators of early LR development, crucial for LR emergence and outgrowth. Although the T-DNA single mutants *plt3*, *plt5*, and *plt7* do not have an obvious phenotype, *plt3plt7* double and *plt3plt5plt7* triple mutants almost completely lack emerged LRs in the first two weeks of development [13]. Examination of the LR founder cells showed defects in cell division indicating that PLTs are responsible for stem cell maintenance and establishment of de novo meristems [14]. The partial redundancy of these three *PLT* genes provides an opportunity to unravel their sensitive dependencies on shaping RSA. 

In this study, we compared the RSA of Arabidopsis plants grown on agar-based medium and potting soil. For this purpose, we generated and studied CRISPR mutants of the redundant PLT transcription factor family. We revealed that the RSA of *plt* CRISPR mutants responded in a genotype-specific manner to plates and rhizotrons by analyzing overall shape and individual traits. While both systems were able to capture subtle and conspicuous phenotypes, the observed genotype dependency emphasizes that neither growth system should be discarded in favor of the other without careful considerations.

## 2. Materials and Methods

### 2.1. Plant Material and Constructs

*plt3*, *plt5*, and *plt7* single mutants were constructed through CRISPR/Cas9 gene editing using one sgRNA per gene (Table S1). Gene-editing constructs were generated using golden gate cloning [15,16]. Unless otherwise stated, plasmids originated from the MoClo Tool kit and Plant Parts kit (Addgene #1,000,000,044, #1,000,000,047). *pAGM4723-FASTR-RPS5a::aCas9-PLTx_sgRNA* constructs were generated by using corresponding spacer sequences indicated in Table S1 to design forward sgRNA primers. These were used to amplify *PLT3sgRNA*, *PLT5sgRNA*, and *PLT7sgRNA* from the *pICH86966::AtU6p::sgRNA_PDS* template construct (Addgene plasmid 46966). PCR products were combined with AtU6-26 promoter from level 0 plasmid *pICSL90002* (Addgene plasmid 68261) into level 1 vectors *pICH47751*. Subsequently, level 1 vectors harboring sgRNAs were combined with *pICH47732-FAST_R* (RFP seed selection [17]), *pICH47742-RPS5A::aCas9*, and the end linker *pICH41766* into the level 2 binary vector *pAGM4723*. *pICH47732-FAST_R* was generated by golden gate cloning of the *pFAST-R* selection cassette from *pICSL7008* (monomeric tagRFP from *Entacmaea quadricolor* fused to the coding sequence of *AtOLE1*) into *pISCH47732*. *pICH47742-RPS5A::aCas9* was generated by assembling *pICH41233-RPS5A*, *pICH41308-aCas9*, and *pICH41421(nosT)* into *pICH47742*. The *RPS5A* promoter was amplified using pRPS5AF-BpiGGAG and pRPS5AR-BpiTACT (Table S1) followed by golden gate cloning into *pICH41233*. A plasmid harboring the Arabidopsis codon optimized *aCas9* was kindly provided by the Puchta lab [18] and amplified using aCas9F-BpiAATG and aCas9R-BpiGCTT (Table S1) followed by golden gate cloning into *pICH41308*.

Transgenic plants were obtained by *Agrobacterium tumefaciens* (C58C1.pMP90)-mediated transformation into wild-type Arabidopsis Col-0. T1 seeds carrying the CRISPR/Cas9 construct were selected under a fluorescence binocular for RFP expression [17], and plants with gene-editing events were transferred to soil, after which inflorescences were genotyped with gene specific primers (Table S1). T1 plants with gene-editing events and transgene-free T2 seeds were selected through RFP selection and genotyped for homozygous gene-editing events. *plt3plt5* was generated by transforming homozygous *plt5-cr* with the *PLT3* CRISPR construct. *plt3plt7* was generated by crossing homozygous *plt3-cr1* and *plt7*-*cr1* and selfing. *plt3plt5plt7* was generated by transforming *plt3plt5* (*plt3-cr1*, *plt5-cr*) with the *PLT7* CRISPR construct. *plt5plt7* was generated by backcrossing *plt3plt5plt7* (*plt3-cr1, plt5-cr, plt7-cr2)* with Col-0 and selfing the F2. The *shr-2* [19] and *scr-4* [19] transgenic lines were described previously.

### 2.2. Plant Growth Conditions and Experimental Set-Up

Seeds were gas sterilized (4% sodium hypochlorite solution mixed with 3 mL 37% HCl) and subsequently plated on square Petri dishes (12 × 12 cm) containing ½ Murashige and Skoog (MS) medium including vitamins (Duchefa, M0222.0050) and 1% agar (Duchefa, P1001.1000). Plates were placed for 3 days in dark at 4 °C for stratification. After stratification, plates were positioned vertically in a growth chamber with 16 h light (50 μmol·m^−2^·s^−1^) 23 °C and 8 h dark 21 °C cycles to germinate. At 5 days post germination (dpg), seedlings were transferred to either the plate or the rhizotron system, where seedlings continued to grow for 15 days. Both systems were placed in the growth chamber with the settings described above. For the plate system, 12–17 replicates (individual seedlings), and for the rhizotron system, 5–8 replicates (individual seedlings) were grown (Table S2). Replicates of both systems were divided over three different experiments with identical conditions.

The plate system consisted of large square Petri dishes (30 × 30 cm^2^) containing ½ MS including vitamins and 1% agar. At 5 dpg, seedlings were transferred to the large plates, after which they were closed with micropore tape (Duchefa). Per plate two seedlings of different genotypes were grown. The plates were positioned vertically in metal racks in randomized groups of five. At 10 dpg, the excess liquid accumulating in the bottom of the plate was removed.

The rhizotron system was a flat box comprising two plastic sheets with a space of 4 mm filled with potting soil (Lensli). A detailed protocol on the rhizotron assembly and preparation can be found in Data S1 and Figure S1. At 5 dpg, three seedlings of the same genotype were planted in each rhizotron, which were then positioned in boxes under a 43° angle in randomized groups of four. The top of the rhizotron was closed with Saran wrap, which was removed at 9 dpg. At 9 dpg and 11 dpg, (poorer growing) seedlings were removed leaving one (best growing) seedling per rhizotron for the remainder of the experiment. Rhizotrons were watered at 9, 11, 14, 16, and 18 dpg with 5 to 10 mL to saturate the topsoil layer (~2 cm). The rest of the soil remained at the same moisture level throughout the experiment. 

### 2.3. Data Acquisition

Twenty days post germination, root systems were imaged using both reflective and contrast scanning. The reflective scans provided a true image of the shape of the root systems in their environment but were not suitable for trait-based analysis due to the complex and overlapping root systems on the plates and poor visibility of each individual root in the rhizotrons. The contrast scans provided details on the different RSA traits. All scans were done with a tabletop flatbed scanner (Epson Expression 11,000 XL) including an A3 transparency unit. 

First, root systems were scanned in the original environment (either plate or rhizotron) using reflective scanning (48-bit color and 800, 1000, or 1200 dpi). For contrast scanning, root systems were collected and separated from the rosette at the hypocotyl. Root systems grown in rhizotrons were gently washed with tap water to remove debris. The isolated root systems were placed in a transparent tray with a thin layer of water to spread them out in a single plane. Contrast scans were made by scanning with the transparency unit, a two-sided light source (16-bit grayscale and 800 or 1200 dpi). 

### 2.4. Data and Statistical Analysis

Root systems were analyzed using a trait-based and shape-based approach. Replicates with obvious growth defects or irreparable technical issues (i.e., too many soil particles, unidentifiable primary root, etc.) were excluded from the analysis.

For the shaped-based approach, reflective scans were analyzed using geometric morphometrics, as described in Klingenberg (2011) [20] and Aceves-García et al. (2016) [21]. Per root system, five landmarks were placed using TpsDig2 software [22]: the center of the rosette (1), the two widest points of the system (2 and 3), the tip of the lowest root (5), and the emergence position of the final secondary root on the lowest root (4). Landmarks 2 and 3 were defined respective to the axis determined by drawing a straight line through landmark 1 and 5. In the occasion a root tip bended by touching the bottom of the plates, a manual correction was made to obtain the predicted shape of the root system. Bent root tips were observed across various genotypes. A description of this correction is provided in Data S2 and Figure S2. MorphoJ software was used to apply least-squares Procrustes superimpositions. Superimposed, normalized shapes were used to compute principal component (PCA) and canonical variate (CVA) analyses. Significance between group means was calculated by performing 10,000 permutation tests on the Mahalanobis distance (* *p* < 0.05; ** *p* < 0.01; *** *p* < 0.001). The PCA and CVA data was exported to R [23] and plotted with ggplot2 [24].

For the trait-based approach, contrast scans were analyzed using the ImageJ plugin SmartRoot [25] and WinRhizo image analysis (WinRhizo Pro 2017a, Regent Instruments Canada Inc.). With SmartRoot, primary root length (cm), number of emerged secondary roots (n; secondary roots with length > 200 μm), and emergence position (cm) of secondary roots on the primary root were measured. Broken primary roots were digitally repaired if possible and otherwise discarded. A description of this correction is provided in Data S2 and Figure S3. The total root length (cm) of the root systems was measured with WinRhizo, except for *shr-2* and *scr-4,* of which the total root length was determined with SmartRoot. Based on the measurements of both SmartRoot and WinRhizo, the total side root length (cm; total root length–primary root length), branching zone (cm; first emergence position–last emergence position), relative branching zone (%; branching zone as percentage of primary root), secondary root density (n/cm; n secondary roots/cm primary root), and interbranch length (cm; lengths below 50 μm were set to 0) per root system were calculated. Subsequent data analyses were performed in R. Plots were made with the package ggplot2. Statistical comparisons between plates and rhizotrons were made by using pairwise *t*-tests and Mann–Whitney U tests depending on whether the data was normally distributed as determined by a Shapiro–Wilk test. Further distinctions were made in *t*-tests (i.e., Welch’s *t*-test or Student’s *t*-test) as determined by Bartlett’s test of homogeneity of variance. Statistical comparisons of all genotypes with Col-0 were performed identically, but also including a Bonferroni correction for multiple testing. All tests were performed in a two-tailed manner with exception of the pie charts displaying the relative contributions, which were performed in a one-tailed manner. Significance is indicated as follows: * *p* < 0.05; ** *p* < 0.01; *** *p* < 0.001. 

## 3. Results

### 3.1. plt CRISPR Mutants Resemble Described T-DNA Lines

**Figure 1:**
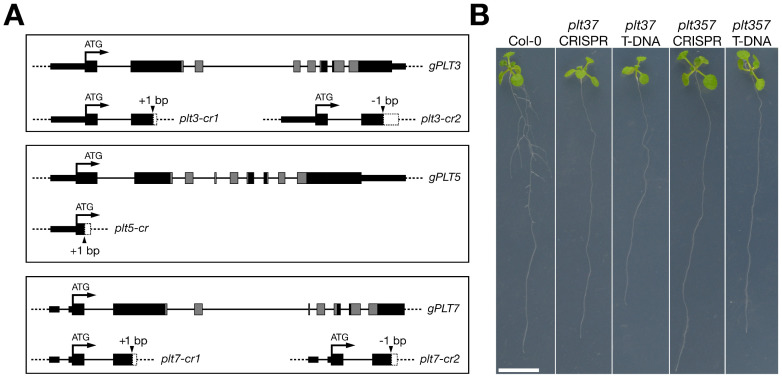
Secondary root development was compromised in *plt3plt7-cr* and *plt3plt5plt7-cr*. (**A**) Gene models of g*PLT3*, g*PLT5*, and g*PLT7* and the locations of the CRISPR indels. Thick black rectangles represent exons, thin black rectangles represent UTR regions, and black lines represent introns. In each model, the AP2 domains are indicated in grey. Arrows point to insertion/deletion sites. White rectangles show out-of-frame amino acids after an indel up to premature stop codons. Gene models shown are isoforms AT5G10510.3 (*gPLT3*), AT5G57390.1 (*gPLT5*), and AT5G65510.1 (*gPLT7*). (**B**) Twelve-day post germination Col-0, *plt3plt7*, and *plt3plt5plt7* mutants on agar plates. Representative individuals are shown for both T-DNA and CRISPR lines. Scale bar corresponds to 1 cm in all photographs.

We previously described *plt3*, *plt5*, and *plt7* single mutants and all corresponding higher-order mutant phenotypes based on T-DNA insertion lines [13]. In order to study the RSA in the same genetic background of transgene-free *plt* knockout lines, we employed CRISPR/Cas9 technology to generate frame shifts in front of the AP2 domains that are required for DNA binding and thus PLT function. For all three *PLT* genes, at least one indel CRISPR line (*plt-cr*) was obtained in the T1 generation, which upon selfing yielded transgene-free homozygous *plt3*, *plt5*, and *plt7* single mutants (Figure 1A; Table S3). In order to produce all possible higher-order mutants, we used a combination of transformation, crossing, and selfing of the various *plt-cr* single mutants (see Materials and Methods). In total, this gave rise to seven mutant lines (Table S4).

In order to assess whether *plt-cr* phenotypes resembled those of the T-DNA insertion lines, we grew each *plt-cr* mutant alongside its T-DNA counterpart on plates. At 12 dpg, obvious RSA defects could be observed only for *plt3plt7-cr* and *plt3plt5plt7-cr,* conforming to the phenotypes of the corresponding T-DNA combinations (Figure 1B; [13,14]). Both CRISPR and T-DNA mutants of *plt3plt7* and *plt3plt5plt7* exhibited severe defects in the outgrowth of secondary roots (Figure 1B). Other *plt-cr* mutants, similarly to their T-DNA counterparts, showed no evident phenotypic defects when compared to wild type plants (Figure S4). We thus demonstrate that our collection of *plt-cr* (henceforth *plt* in this study) mutants behaved in accordance with previously described phenotypes with respect to RSA.

### 3.2. RSA Shape of plt3plt7 and plt3plt5plt7 Was Distinct from Other Genotypes in Plates and Rhizotrons

**Figure 2:**
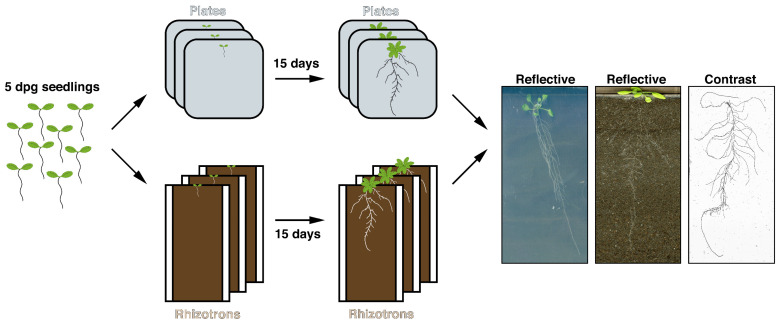
Experimental setup for studying the responses of plates and rhizotrons on RSA. Scans shown are representative of reflective and contrast scans.

Up until now, root architecture mutants have mainly been identified by using various mutagenesis approaches coupled with extensive screening of these mutants on agar-based plates. This in vitro plate method, however, does not reflect the natural situation plants normally grow in. One could therefore question whether the plate system harbors the best possibilities to obtain all relevant RSA phenotypes, especially in a more natural context. We addressed this question by performing growth assays in an agar-based system (plates) versus a soil-based system (rhizotrons) with the abovementioned *plt* CRISPR mutants. To this end, we developed an experimental set-up that allowed for a detailed comparison using a dual approach combining both overall RSA shape and individual traits constituting RSA (Figure 2).

**Figure 3:**
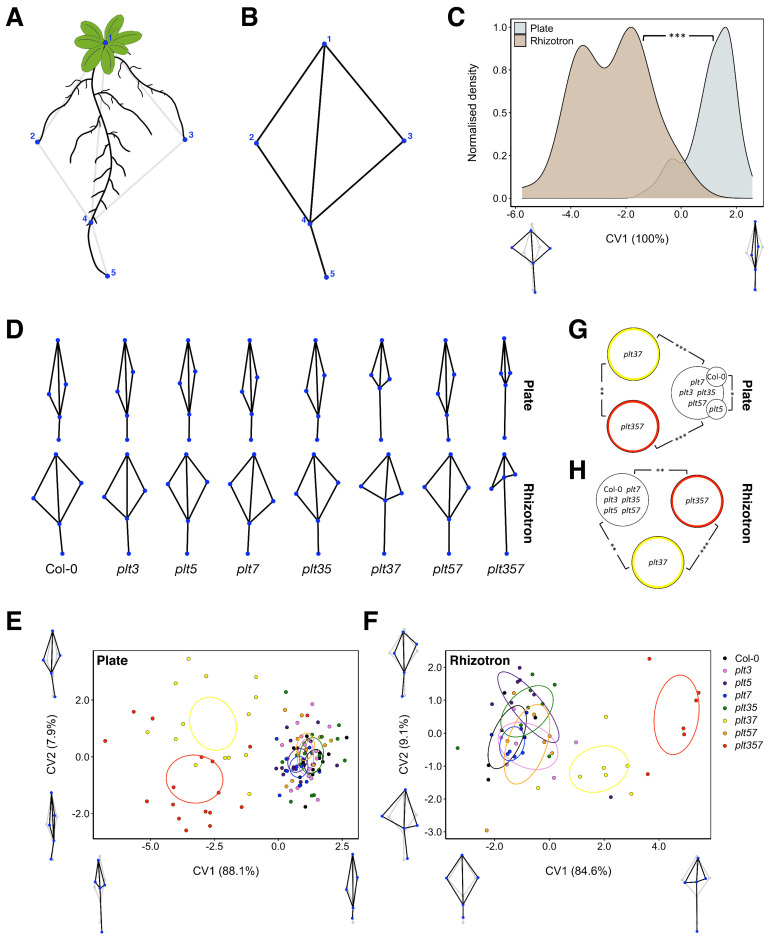
Variations in *plt* RSA shape in plates and rhizotrons were captured by geometric morphometrics. (**A**) Schematic representation of the position of the five landmarks used for the geometric morphometric analysis. (**B**) Morphometric wireframe based on the five landmarks. (**C**) CVA of RSA shape comparing plates with rhizotrons for all genotypes combined. Small wireframes represent the shapes at the limits of the CV1 axis (black/blue), with in grey the shape at CV1 = 0. Significance asterisks indicate RSA shape changes based on the Mahalanobis distance. (**D**) Morphometric wireframes for each genotype on plates and rhizotrons. (**E**) CVA of RSA shape comparing genotypes in plates. (**F**) CVA of RSA shape comparing genotypes in rhizotrons. In (**E**,**F**), 95% confidence ellipses of the mean are indicated. (**G**) Diagram displaying significant RSA shape differences between genotypes on plates, based on Mahalanobis distances. (**H**) Diagram displaying significant RSA shape differences between genotypes in rhizotrons, based on Mahalanobis distances. * *p* < 0.05; ** *p* < 0.01; *** *p* < 0.001.

We first assessed variations in overall shape change for each growth system and genotype by performing a geometric morphometric analysis using high-resolution reflective scans. This approach has previously been used to reveal shape differences in Arabidopsis ecotypes [21]. It exploits the information provided by defined points on a root system—landmarks—to make coordinate-based shapes for each plant, which are then superimposed and normalized to make comparisons between experimental groups. We defined five landmarks that could be discerned well for all genotypes in both plates and rhizotrons: the center of the rosette (1), the two widest points of the system (2 and 3), the tip of the lowest root (5), and the emergence position of the final secondary root on the lowest root (4) (Figure 3A,B). 

We asked whether the overall shape on plates and rhizotrons was different by pooling all genotypes. From a canonical variate analysis (CVA), which maximizes the variance between predefined groups while minimizing the within-group variance, it becomes evident that the difference between rhizotron and plate shapes was extremely significant (Figure 3C). Specifically, the RSA in rhizotrons was evidently wider than that observed in plates (Figure 3C). A principal component analysis (PCA) revealed, however, that most variation (PC1 44.3% and PC2 22.5%) in our dataset originated from the y-coordinates of the widest points (landmark 2 and 3), which did not separate plates and rhizotrons well (Figure S5A). A clear separation can only be seen in PC3 (17.1%) and particularly PC4 (12.4%), which correspond to the distance between landmarks 4 and 5, and system width, respectively (Figure S5B,C).

We then investigated whether certain *plt* mutants possessed divergent RSA shapes for plates and rhizotrons separately (Figure 3D). In both plates (Figure 3E,G) and rhizotrons (Figure 3F,H), we observed highly significant differences between *plt3plt7*, *plt3plt5plt7*, and the other genotypes. In both growth systems, the distance between landmarks 4 and 5 (CV1) was markedly greater in *plt3plt7* and *plt3plt5plt7* mutants compared to the rest, which was also reflected in principal components explaining variance related to this feature (Figure S6). *plt3plt7* and *plt3plt5plt7* shapes were significantly different from each other as well (Figure 3G,H). In plates and rhizotrons, *plt3plt7* plants grew wider than *plt3plt5plt7* mutants. Moreover, *plt3plt7* mutants grown in rhizotrons had a smaller distance between landmarks 4 and 5 than *plt3plt5plt7* mutants, which was not observed in plates (Figure 3D–F). We also found a significant difference between Col-0 and *plt5* RSA shape in plates on CV1, albeit a smaller one than that described for *plt3plt7* and *plt3plt5plt7* (Figure 3E,G). 

In conclusion, the shape of RSA is clearly affected by the type of growth system used, shown by a dramatic increase in RSA width when the same genotypes were grown on rhizotrons versus plates. Nevertheless, despite using different growth systems, *plt3plt7* and *plt3plt5plt7* mutants can be consistently separated based on similar variations in landmark positioning. In terms of biology, agar-based and soil substrates seem both perfectly suitable to study the RSA shape effects on *plt* mutants.

### 3.3. RSA Traits Differed between Plates and Rhizotrons, and Responses Were Genotype Dependent

With the shape-based approach, we were able to detect genotypes that were in overall shape different from one another within plates and rhizotrons. However, RSA is vastly more complex than a shape, consisting of many individual traits that combined define the architectural state of a root system. Accordingly, we also employed a trait-based approach to bring more subtle differences to light by using high-resolution contrast scans. 

**Figure 4:**
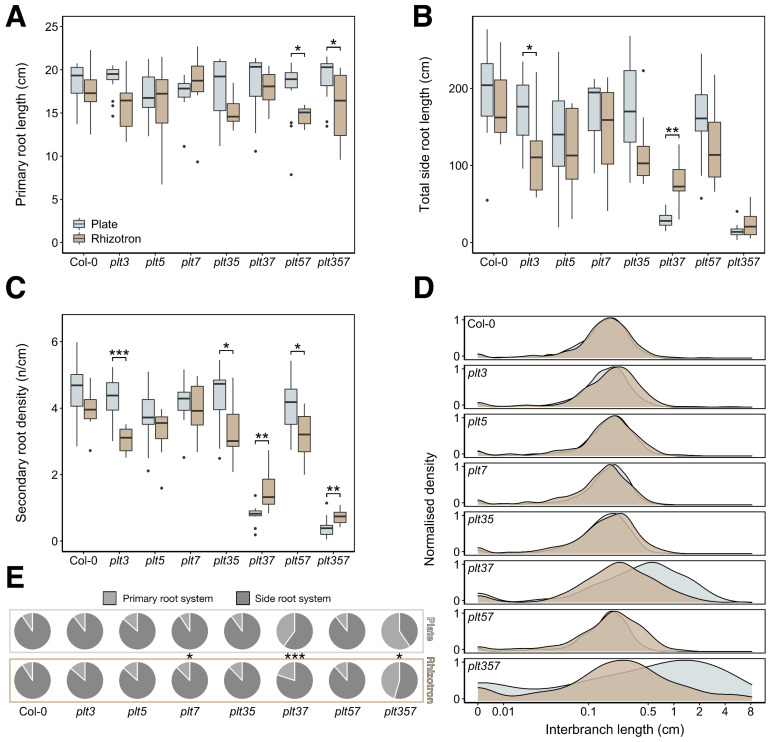
Various RSA traits of Col-0 and *plt* mutant combinations in plates and rhizotrons. (**A**) Primary root length per genotype. (**B**) Total side root length per genotype. (**C**) Secondary root density per genotype. (**D**) Normalized density plots of interbranch length per genotype. (**E**) Pie charts displaying the relative contributions of the primary root and the side roots to the total root length. Significance asterisks denote pairwise comparisons between plate and rhizotron values for each genotype. * *p* < 0.05; ** *p* < 0.01; *** *p* < 0.001.

When grown in rhizotrons, all genotypes except *plt7* tended to have either an unchanged or slightly shorter primary root length than observed in plates, although this reduction was only significant for *plt5plt7* and *plt3plt5plt7* (Figure 4A). Likewise, the total side root length appeared somewhat reduced in most genotypes, with the exception of *plt3plt7* and *plt3plt5plt7* (Figure 4B). Contrarily, these two severely compromised mutants exhibited an increase in total side root length in rhizotrons. The variation in total side root length likely resulted from differences in the number of emerged secondary roots (Figure S7B). By expressing this number relative to the primary root length to correct for differences in the latter, yielding the secondary root density, the observed trends are even more conspicuous (Figure 4C). We identified a highly significant increase in secondary root density in *plt3plt7* and *plt3plt5plt7* in rhizotrons, whereas it was significantly decreased in *plt3*, *plt3plt5*, and *plt5plt7*. To identify how this increased density was established, we investigated the distribution of interbranch length (i.e., the distance between two consecutive secondary roots) across the primary roots of each genotype. The distribution of interbranch lengths was strongly shifted in *plt3plt7* and *plt3plt5plt7* but not altered in overall shape, demonstrating that these two genotypes simply produced emerged secondary roots at shorter intervals in rhizotrons (Figure 4D). For *plt3*, *plt3plt5*, and *plt5plt7*, a very slight shift was observed in the opposite direction, which is in line with their significantly decreased emerged secondary roots in rhizotrons, although their branching zone length was also reduced (Figure S7C). Other measured traits, including the total root length and the size of the relative branching zone, reflected the earlier observations that except for *plt3plt7* and *plt3plt5plt7,* genotypes tended to have a less developed root system in rhizotrons compared to plates (Figure S7A,C,D). The pronounced phenotypes of *plt3plt7* and *plt3plt5plt7* also stand out when comparing each genotype with Col-0, since significant differences can be found only for these two mutants in both plates and rhizotrons (Figure S8). Notably, the differences between Col-0 and *plt3plt7* and *plt3plt5plt7* seem more pronounced in plates compared to in rhizotrons (Figure S8).

We then asked what the apparent increases and decreases in secondary and side root traits implied for the total RSA of each genotype. By calculating the contribution of the primary and side root systems to the total root system, we identified a significant increase in the contribution of the side root system for *plt3plt7* and *plt3plt5plt7* in rhizotrons of roughly 15 to 20 percent (Figure 4E). This is in accordance with their phenotypes on rhizotrons consisting of of significantly higher secondary root density and corresponding decreased interbranch length while having a shorter primary root. Thus, we demonstrate that RSA traits can significantly differ between growth systems, and importantly, that the effect of these systems is genotype dependent in *plt* mutants.

### 3.4. Differential RSA Responses to Plates and Rhizotrons Not Limited to PLT Gene Family

To unravel whether the observed genotype-dependency responses of the *plt* mutants to plates and rhizotrons could also be observed outside of this gene family, we conducted the same approach using *shr-2* and *scr-4* T-DNA mutants. SCARECROW (SCR) and SHORT-ROOT (SHR) are two transcription factors belonging to the GRAS family that control the asymmetric division of the ground tissue stem cell daughter that generates cortex and endodermis in the root meristem [26,27,28,29]. Beside their role in primary root development, SHR and SCR are both required for lateral root initiation and patterning of lateral root primordia. In addition, SHR and SCR are required for maintaining the indeterminate growth of primary, lateral, and adventitious roots [30,31,32]. This indicates that these mutants have severe root developmental defects which are completely different from the *plt3*, *plt5*, and *plt7* and their corresponding higher order mutants.

**Figure 5:**
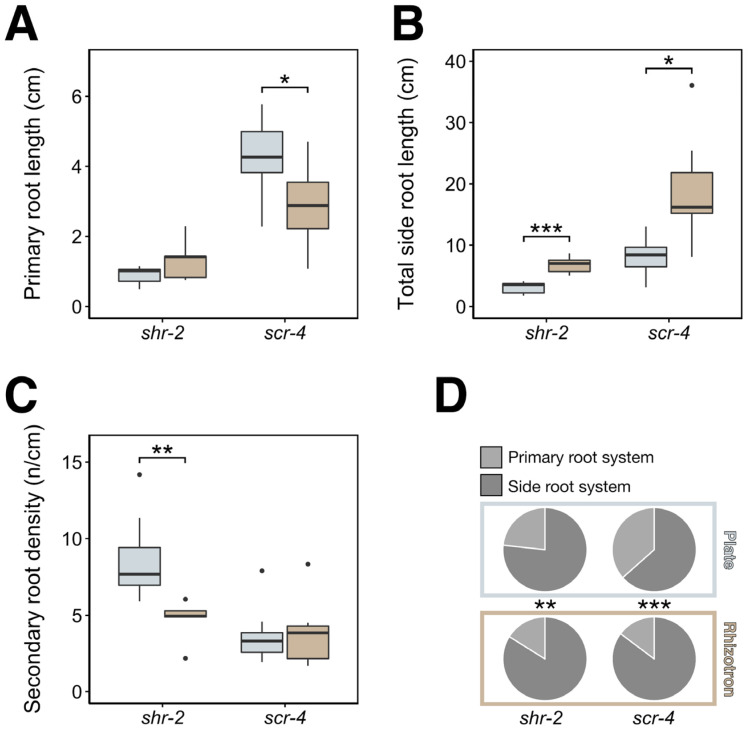
Various RSA traits of *shr-2* and *scr-4* mutants in plates and rhizotrons. (**A**) Primary root length per genotype. (**B**) Total side root length of per genotype. (**C**) Secondary root density per genotype. (**D**) Pie charts displaying the relative contributions of the primary root and the side roots to the total root length. Significance asterisks denote pairwise comparisons between plate and rhizotron values for each genotype. * *p* < 0.05; ** *p* < 0.01; *** *p* < 0.001.

When grown in rhizotrons, *shr-2* had a similar primary root length as in plates, but *scr-4* showed a significantly reduced primary root length, which highly resembles the growth pattern of *plt3plt7* and *plt3plt5plt7* mutants (Figure 5A). The total side root length of both *shr-2* and *scr-4* was significantly larger in rhizotrons, as was the related total root length (Figure 5B and Figure S7). Observations in other root traits, however, highlight the dissimilarities between these two mutants. On one hand, *shr-2* had a significant decrease in secondary root density (Figure 5C), while the number of secondary roots and (relative) branching zone showed only a tentative decrease (Figure S9B–D). On the other hand, *scr-4* had no changes in secondary root density (Figure 5C) but a significantly decreased secondary root number and (relative) branching zone (Figure S9B–D). These opposite results were likely caused by the unchanged and significantly decreased primary root length for *shr-2* and *scr-4*, respectively (Figure 5A).

Although the phenotypes of *shr-2* and *scr-4* do not correspond completely to *plt3plt7* and *plt3plt5plt7*, it is worthwhile to note that all four mutants showed an increase in total side root length on rhizotrons (Figure 4B and Figure 5B). We therefore wondered whether this increase corresponded to an increase in secondary density as in *plt3plt7* and *plt3plt5plt7.* However, we rather observed a significant reduction in secondary root density in *shr-2* and no apparent change in *scr-4* (Figure 5C). Our findings imply that the increase of the total side root length, unlike in *plt3plt7* and *plt3plt5plt7*, originated from increased secondary root length or higher-order branching. This notion is fortified by the fact that interbranch length was not convincingly smaller in rhizotrons than in plates (Figure S9E), as was observed for *plt3plt7* and *plt3plt5plt7*. Nonetheless, *shr-2* and particularly *scr-4* showed a similar significant increased investment in the side root system just like *plt3plt7* and *plt3plt5plt7* (Figure 5D).

In conclusion, the trait-based analysis with *shr-2* and *scr-4* indicates that the genotype dependency of the RSA response to plates and rhizotrons is not limited to the PLT family. Strong parallels were drawn between the phenotypic responses to the growth systems of *plt3plt7*, *plt3plt5plt7*, *shr-2*, and *scr-4* mutants, although the developmental cause of the increased total side root length is different in nature.

## 4. Discussion

In this study, we directly compared the RSA of a collection of *plt* CRISPR mutants between agar-based plates and potting soil rhizotrons. We evaluated overall RSA shape properties (Figure 3, Figures S3 and S4) and detailed individual traits (Figure 4, Figures S5 and S6) by combining reflective and contrast scanning approaches. It was revealed that there is a strong genotype-specific response of *plt* mutants to agar-based and soil substrates. When grown on rhizotrons, most *plt* mutants showed mild to moderate reductions of various RSA traits. The secondary root density of *plt3*, *plt3plt5*, and *plt5plt7* mutants was significantly reduced in rhizotrons, more strongly than Col-0, *plt5*, and *plt7*. However, *plt3plt7* and *plt3plt5plt7* mutants exhibited a contrasting phenotype in rhizotrons, with a higher secondary root density and consequently a more extensive side root system (Figure 4 and Figure S5). This genotype-dependent response is not limited to the *PLT* gene family, as was confirmed by analyzing the RSA of *shr* and *scr* mutants (Figure 5 and Figure S7). 

Apart from comparing growth systems, our dual approach also allowed us to directly compare shape-based and trait-based approaches. Shape is a considerably easier parameter to study in 20 dpg root systems, because it only requires reflective scans and thus omits laborious root washing and contrast scanning. Moreover, the computational workload of positioning five landmarks is only a fraction of the work demanded by extensive tracing in a trait-based approach. However, the resolution is much lower than a trait-based approach, because many details that make up a shape are lost. Still, we could convincingly separate the RSA shapes of *plt3plt7* and *plt3plt5plt7* from the other genotypes in our dataset, which highlights the usefulness of this approach to study RSA.

Although our comparison of Arabidopsis RSA between agar-based and soil substrates was the most extensive one yet, several of our findings are reinforced by previous studies. Rellán-Álvarez et al. (2015) [33] revealed major changes in the transcriptome of Arabidopsis plants depending on the growth system, which was not limited only to genes involved in light signaling and photosynthesis. This suggests a molecular basis underlying the RSA phenotypes we observed, which seems to involve PLT3, PLT5, and PLT7. Gandullo et al. (2021) [34] observed that the primary root and the total root system of several tomato cultivars is reduced in soil compared to agar-based substrate. Moreover, they demonstrated that soil-grown plants are more resilient to salt stress than plate-grown plants. It thus seems that the complex soil environment somehow alleviated deleterious effects imposed by abiotic stress. Our observation that *plt3plt7* and *plt3plt5plt7* mutants had a higher secondary root density when grown on rhizotrons compared to plates might indicate that genotypic defects can be buffered by cues from the soil, although strongly dependent on the genotype. Nonetheless, *plt3plt7* and *plt3plt5plt7* were still distinct from wild type (Figure S8) in both plates and soil, showing that their plate phenotype persists in rhizotrons to some degree. This finding is in agreement with Jiang et al. (2019) [35], who observed that the phenotype of gel-grown maize seedlings persisted in field-grown mature root systems. The genotype dependency of the response to plates versus rhizotrons has interesting implications for understanding the redundancy of the PLT3, PLT5, and PLT7 in RSA. The fact that *plt3* was the only single mutant that is significantly more compromised in rhizotrons than in plates regarding secondary root density (and total side root length) suggests that PLT3 is more important for normal growth in soil than PLT5 and PLT7 (Figure 4B,C). A similar effect was only obtained when both genes were knocked out in *plt5plt7*. However, the *plt5* mutation combined with the *plt3* mutation in *plt3plt5* did not behave any differently than *plt3*. It is evident, though, that PLT7 function partially overlaps with PLT3, since the *plt3plt7* double mutant showed a dramatic phenotype that was exacerbated when PLT5 was also lost in *plt3plt5plt7*. Why, then, the *plt3plt7* and *plt3plt5plt7* mutants are buffered by the soil, but not *plt3*, *plt3plt5*, or *plt5plt7*, remains enigmatic. Possibly, the severity of the phenotype sensitizes the plant to external signals that can in turn activate certain developmental programs, which could explain why the total root length of *shr-2* and *scr-4* also increased in soil. Growth in rhizotrons could slightly stimulate the development of a type of secondary root that is not (fully) dependent on PLT3, PLT5, and PLT7, which is masked in normal root systems with large numbers of secondary roots. This knowledge gap demands further investigation of the molecular processes that regulate RSA development, which should be addressed in future studies.

## 5. Conclusions

Our findings demonstrate that the type of growing system clearly affects both the RSA shape and its underlying traits. It is fascinating that within a set of redundant genes, the RSA response to the type of growing system is genotype dependent, which indicates that the balance between nature (i.e., genotype) and nurture (i.e., environment) shifts depending on their context. To conclude, we strongly advocate to take careful considerations into account for the choice of growth conditions when designing RSA experiments.

## Data Availability

Not applicable.

## Supplementary Materials

The following are available online at https://www.mdpi.com/article/10.3390/genes12071028/s1, Figure S1: Graphical overview of rhizotron assembly, Figure S2: Example of manual correction of bent root tips for the shape-based approach, Figure S3: Example of manual correction of a broken primary root for the trait-based approach. Figure S4: Overview of 12 dpg *plt* CRISPR and T-DNA seedlings compared to (Col-0), Figure S5: Variations in *plt* RSA shape between plates and rhizotrons, Figure S6: RSA shape variation between genotypes in plates and rhizotrons, Figure S7: Various RSA traits of Col-0 and *plt* mutant combinations in plates and rhizotrons, Figure S8: Various RSA traits of Col-0 and *plt* mutant combinations in plates and rhizotrons, Figure S9: Various RSA traits of *shr-2* and *scr-4* mutants in plates and rhizotrons, Table S1: Primers used in this study. sgRNA spacer sequences are indicated in capitals in the primer, Table S2: Number of replicates per genotype and growth system, Table S3: Mutations underlying the genotypes used in this study. Protein positions are numbered according to the ATG, Table S4: Mutations underlying the single, double, and triple mutants used in this study, Data S1: Detailed rhizotron protocol, Data S2: Description of manual corrections in root architecture analysis.
