# Supplementary material for: Nature and Nurture: Genotype-Dependent Differential Responses of Root Architecture to Agar and Soil Environments
Source: Genes (Basel). 2021 Jul 1;12(7):1028. doi: 10.3390/genes12071028 (PMC8303133; doi:10.3390/genes12071028)
Supplement: Supplementary file 1 [file genes-12-01028-s001.zip › Supplementary-Revised-Latest Version.pdf]

Supplemental data

Table S1 Primers used in this study. sgRNA spacer sequences are indicated in capitals in the primer.

Oligo name	Sequence (5' to 3')	Function
PLT3 sgRNA	tgtggtctcaattGTGGATCGTAGATTTGAGTGgttttagagctagaaatagcaag	CRISPR/Cas9
PLT5 sgRNA	tgtggtctcaattGAGCCAGTTCTGGTGGGAGGgttttagagctagaaatagcaag	CRISPR/Cas9
PLT7 sgRNA	tgtggtctcaattGTAACTCCTTCGGCAACGGTGgttttagagctagaaatagcaag	CRISPR/Cas9
pRPS5AF-BpiGGAG	TGTGAAGACAAGGAGCTCAACTTTTGATTCGCTATTTG	Cloning
pRPS5AR-BpiTACT	TGTGAAGACAAAGTAGGCTGTGGTGAGAGAAACAGA	Cloning
aCas9F-BpiAATG	TGTGAAGACAAAATGGATAAGAAGTACTCTATCGGACTC	Cloning
aCas9R-BpiGCTT	TGTGAAGACAAAAGCTCAAACCTTCCTCTTCTTCTTAGG	Cloning
PLT3 CRISPR F	AGGCGTTCCCATTCTCTTTT	Genotyping
PLT3 CRISPR R	TTGACCAAACGTATCAGCAATC	Genotyping/Sequencing
PLT5 CRISPR F	AGTCTCGACGCTCGTGTTCT	Genotyping
PLT5 CRISPR R	CTTGCCGTTCGACTGAAAAT	Genotyping/Sequencing
PLT7 CRISPR F	GCTCTGTTGTTAGCCGACTT	Genotyping/Sequencing
PLT7 CRISPR R	AATTACCGGGTGACTCCACG	Genotyping

Table S2 Number of replicates per genotype and growth system.

Genotype	# plate replicates	# rhizotron replicates
Col-0	14	6
plt3	15	6
plt5	15	8
plt7	13	6
plt3plt5	14	8
plt3plt7	14	7
plt5plt7	15	7
plt3plt5plt7	17	6
shr-2	13	5
scr-4	12	7

Table S3 Mutations underlying the genotypes used in this study. Protein positions are numbered according to the ATG. Nucleotide positions are numbered according to the TSS. Indel identity is respective to gene orientation.

Gene/Mutant	Protein size (AA)	Start AP2 domain 1 (AA)	Indel (nt)	Indel position (nt)
gPLT3 (AT5G10510.3)	604	268		
plt3-cr1	130		+ T	1283
plt3-cr2	186		- T	1283
gPLT5 (AT5G57390.1)	558	203		
plt5-cr	38		+ A	342
gPLT7 (AT5G65510.1)	498	231		
plt7-cr1	123		+ C	888
plt7-cr2	132		- C	888

Table S4 Mutations underlying the single, double and triple mutants used in this study. Mutations are visualized in Figure 1A and described Table S3.

Mutation(s)	Genotype
plt3-cr1	*plt3*
plt5-cr	*plt5*
plt7-cr1	*plt7*
plt3-cr2, plt5-cr	*plt3plt5*
plt3-cr1, plt7-cr1	*plt3plt7*
plt5-cr, plt7-cr2	*plt5plt7*
plt3-cr1, plt5-cr, plt7-cr2	*plt3plt5plt7*

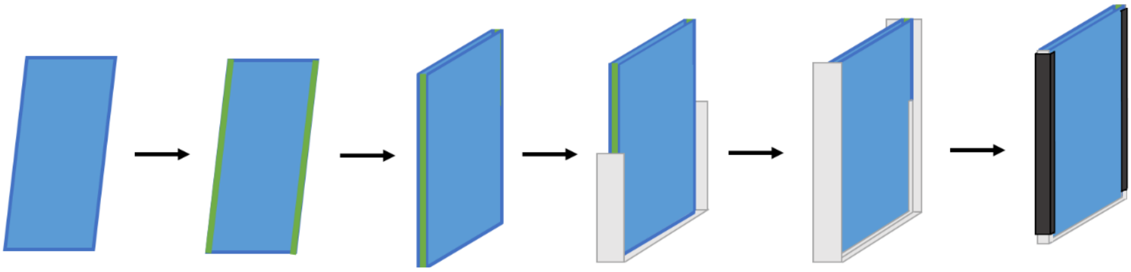

Figure S1 Graphical overview of rhizotron assembly.

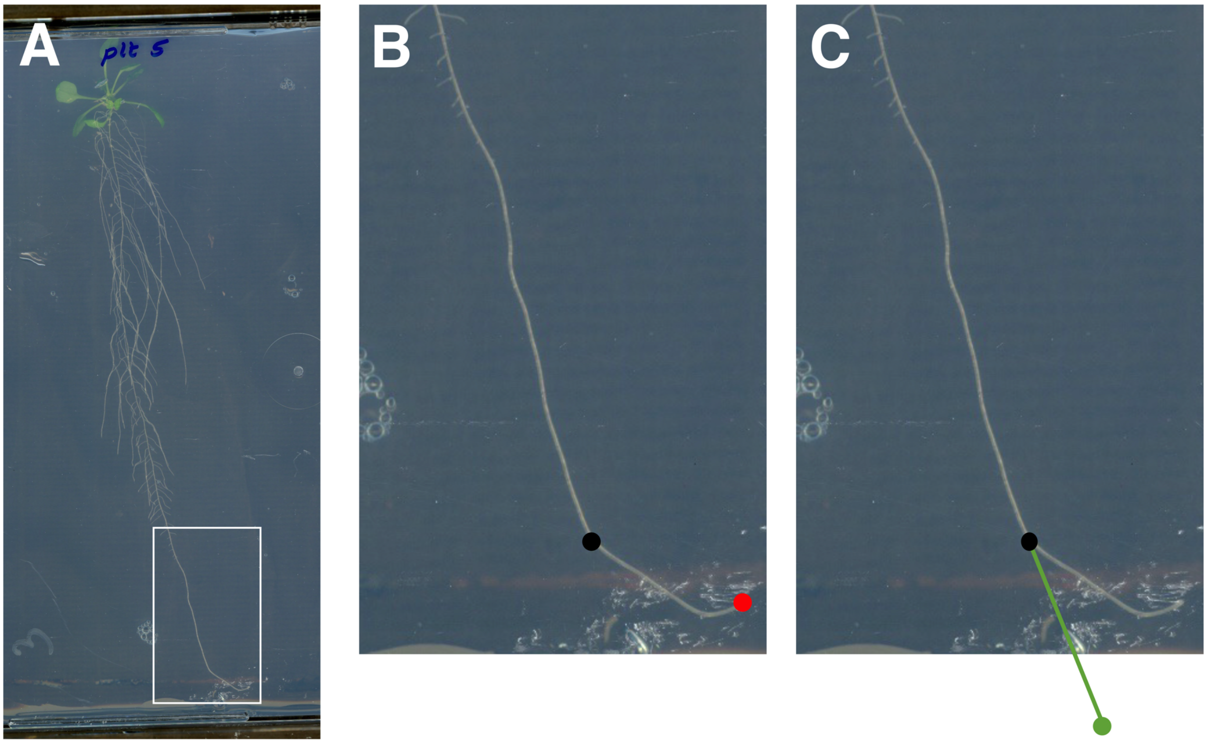

Figure S2 Example of manual correction of bent root tips for the shape-based approach. **(A)** Reflective scan showing bending of a root tip due to space limitations. **(B)** Close-up of the root tip with uncorrected landmark 5. Black dot indicates bending initiation of the root. Red dot indicates the uncorrected position of landmark 5. Length was measured between these points. **(C)** Close-up of the root tip with corrected landmark 5. Black dot indicates bending initiation of the root. Green line indicates the correction. Green dot indicates the corrected position landmark 5.

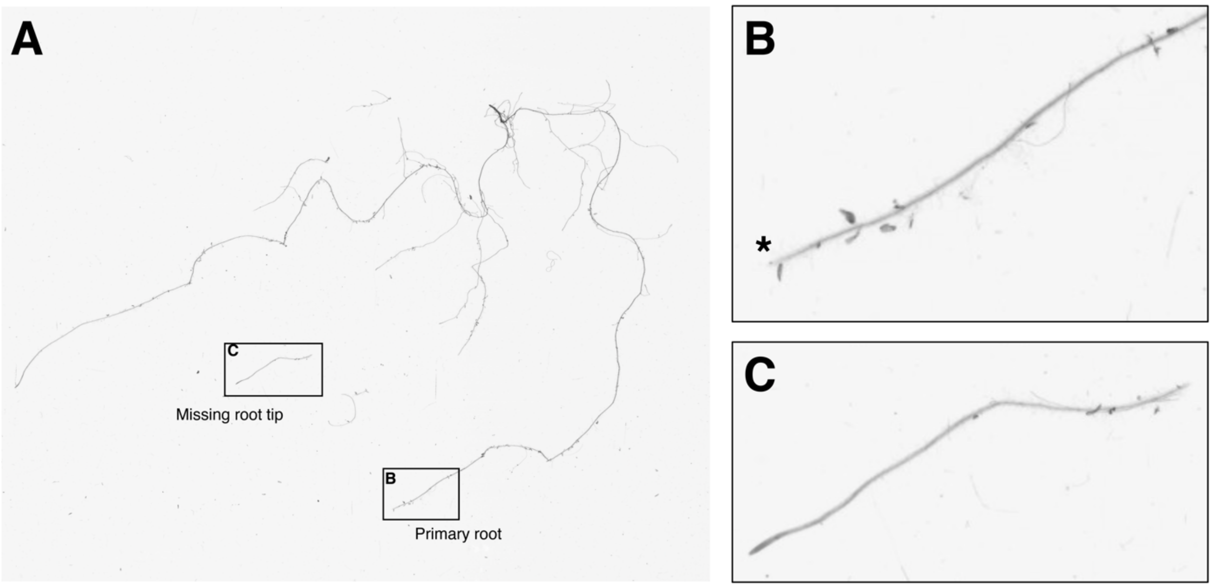

Figure S3 Example of manual correction of a broken primary root for trait-based approach. **(A)** Raw contrast scan showing root system with broken pieces. **(B)** Close-up of broken primary root, note the absence of the root meristem (*). **(C)** Close-up of the remaining part of the primary root.

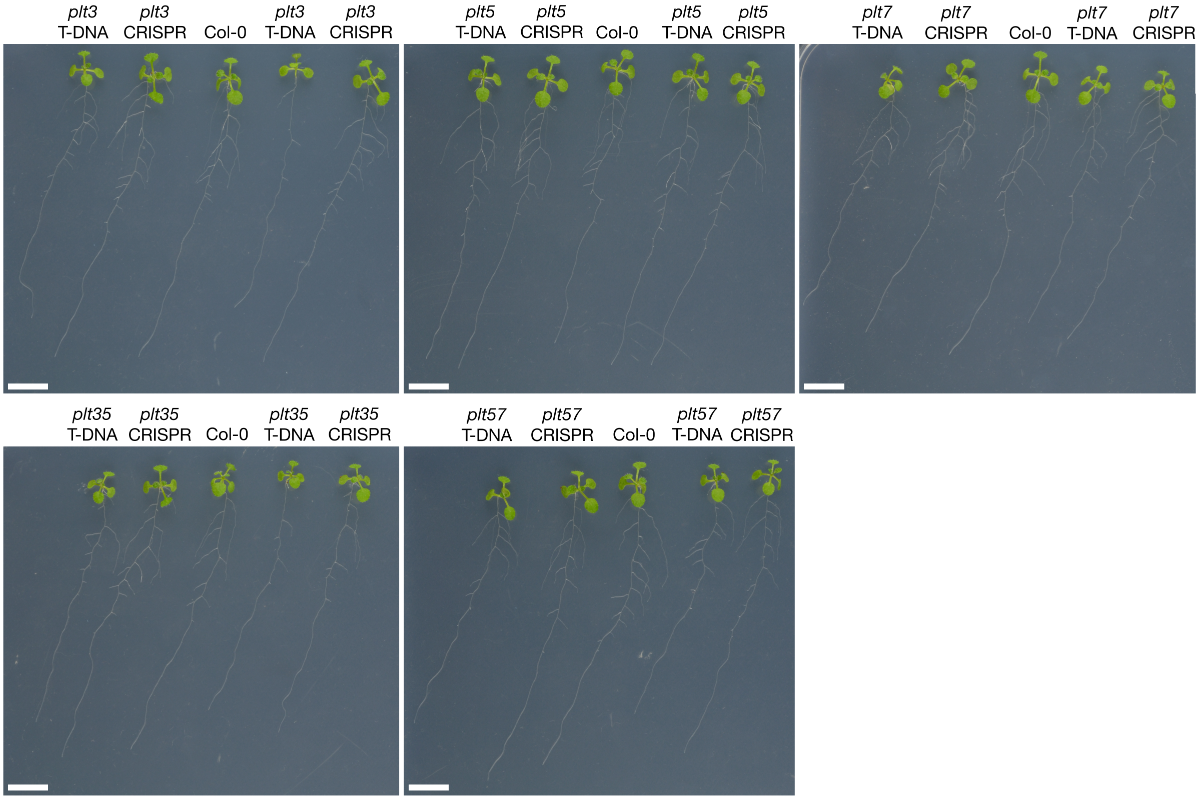

Figure S4 Overview of 12 dpg *plt* CRISPR and T-DNA seedlings compared to (Col-0). Two replicates are shown per mutant line. Scale bar corresponds to 1 cm.

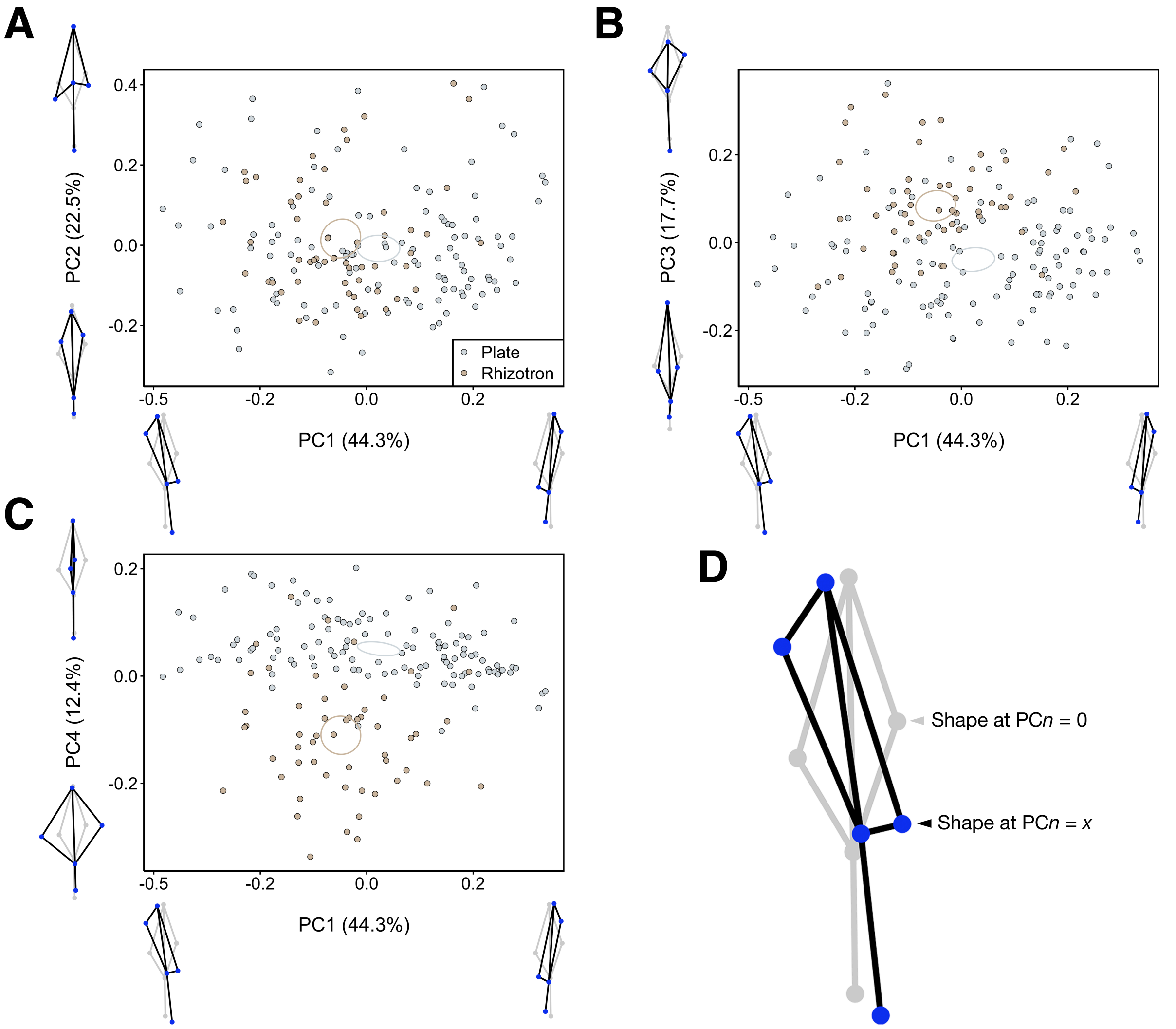

Figure S5 Variations in *plt* RSA shape between plates and rhizotrons. **(A)** PCA of RSA shape of all genotypes combined in both plates and rhizotrons. PC1 and PC2 are displayed. PC3 and PC4 in (**B**) and (**C**), respectively. Small wireframes represent the shapes at the limits of PC axes (black/blue), with in grey the shape at PC*n* = 0. 95% confidence ellipses of the mean are indicated. (**D**) Example morphometric wireframe showing shape change on an arbitrary PC*n* at the values 0 (grey) and *x* (black/blue).

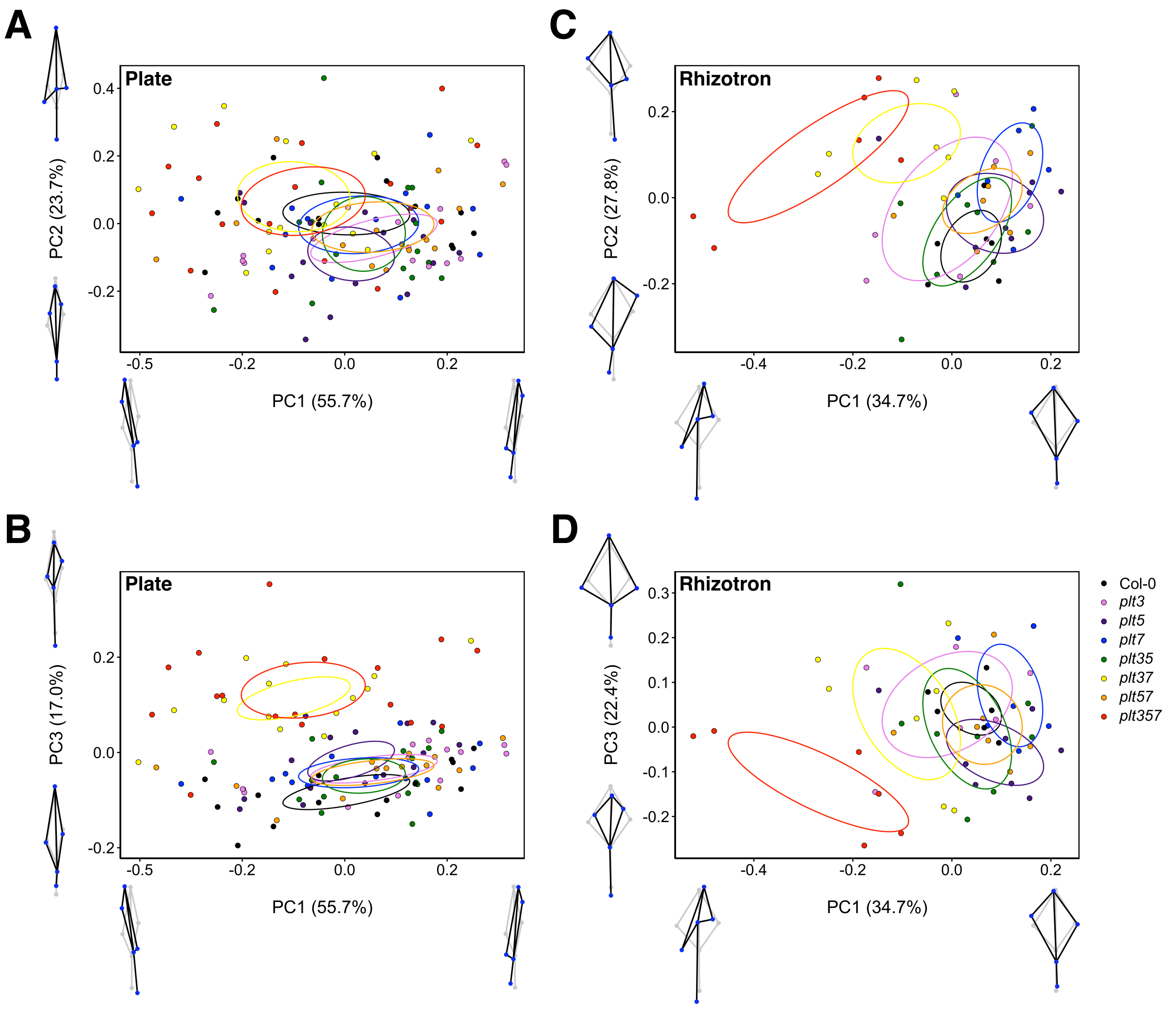

Figure S6 RSA shape variation between genotypes in plates and rhizotrons. (**A**) PCA of RSA shape of Col-0 and *plt* mutant combinations in plates. PC1 and PC2 are displayed. (**C**) PCA of RSA shape of Col-0 and *plt* mutants in rhizotrons. PC1 and PC2 are displayed. PC3 is shown in (**B**) for plates and (**D**) for rhizotrons, respectively. Small wireframes represent the shapes at the limits of PC axes (black/blue), with in grey the shape at PCn = 0. 95% confidence ellipses of the mean are indicated.

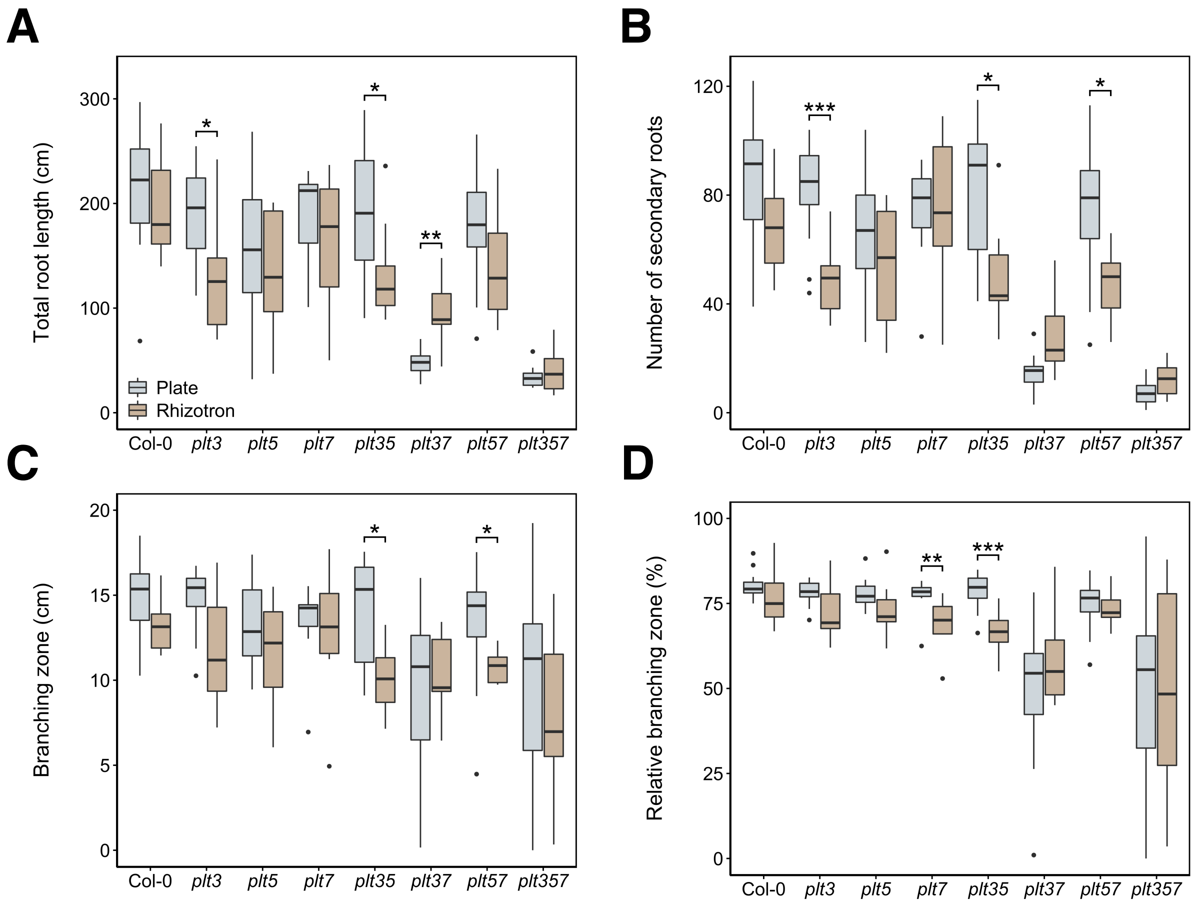

Figure S7 Various RSA traits of Col-0 and *plt* mutant combinations in plates and rhizotrons. (**A**) Total root length per genotype. (**B**) Number of secondary roots per genotype. (**C**) Absolute branching zone per genotype. (**D**) Relative branching zone per genotype. Significance asterisks denote pairwise comparisons between plate and rhizotron values for each genotype. * $p < 0.05$; ** $p < 0.01$; *** $p < 0.001$.

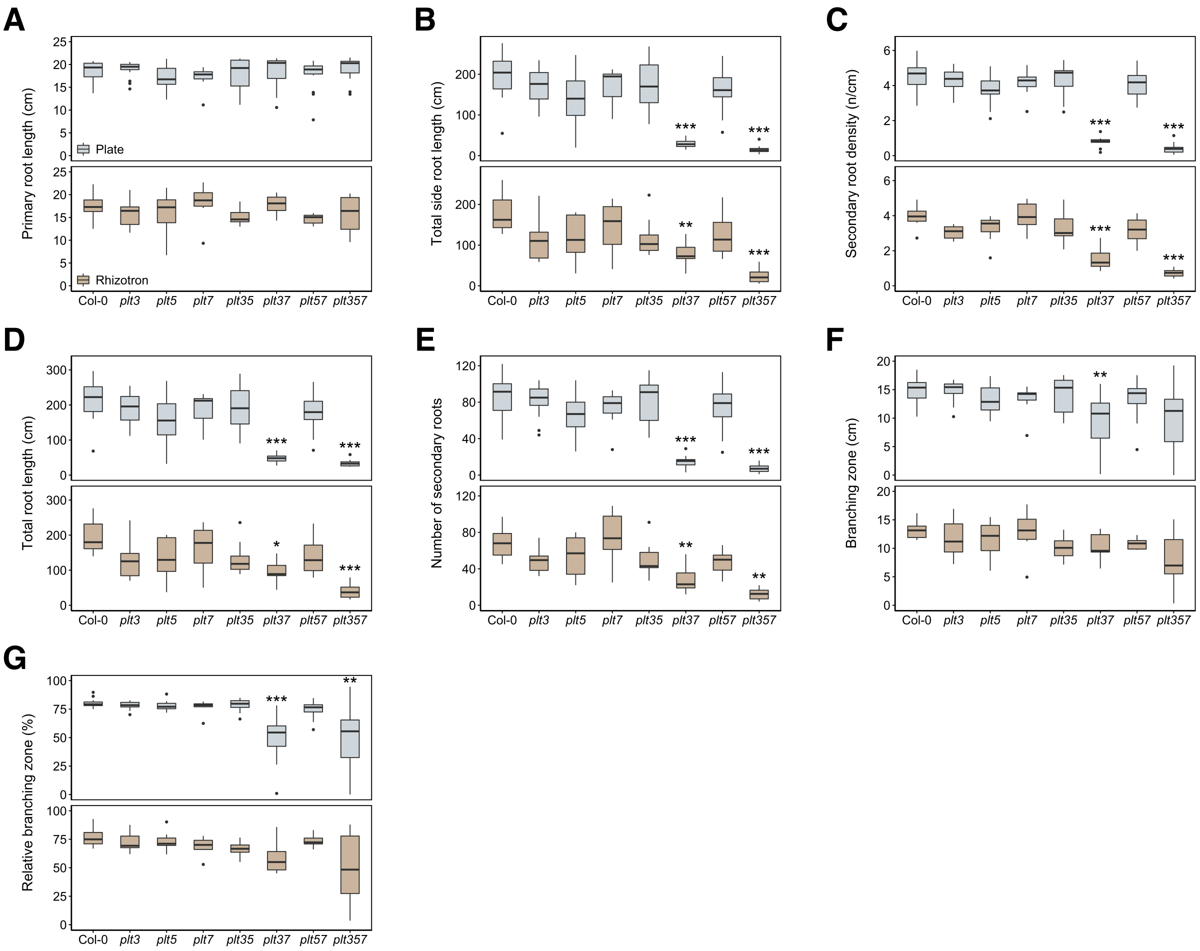

Figure S8 Various RSA traits of Col-0 and *plt* mutant combinations in plates and rhizotrons. (**A**) Primary root length per genotype. (**B**) Total side root length per genotype. (**C**) Secondary root density per genotype. (**D**) Total root length per genotype. (**E**) Number of secondary roots per genotype. (**F**) Absolute branching zone per genotype. (**G**) Relative branching zone per genotype. Significance asterisks denote pairwise comparisons (Bonferroni-corrected) with Col-0. * $p < 0.05$; ** $p < 0.01$; *** $p < 0.001$.

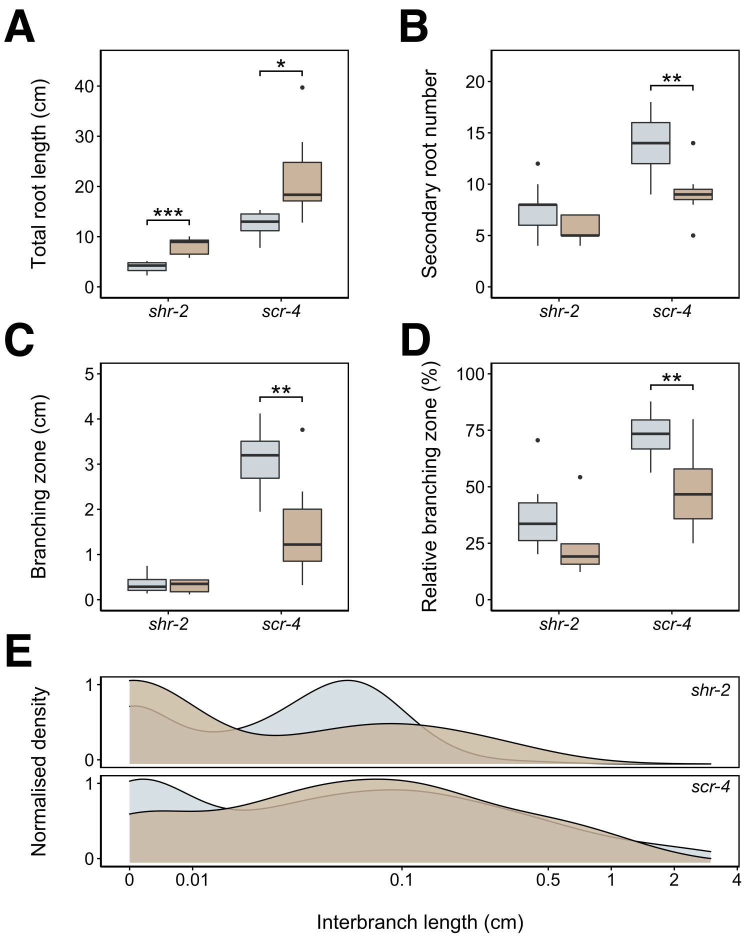

Figure S9 Various RSA traits of *shr-2* and *scr-4* mutants in plates and rhizotrons. **(A)** Total root length per genotype. (**B**) Secondary root number per genotype. (**C**) Absolute branching zone per genotype. (**D**) Relative branching zone per genotype. (**E**) Normalized density plots of interbranch length per genotype. Significance asterisks denote pairwise comparisons between plate and rhizotron values for *shr-2* and *scr-4*. * $p < 0.05$; ** $p < 0.01$; *** $p < 0.001$.

Data S1 Detailed Rhizotron Protocol

For assembly of a rhizotron, two transparent plexiglass plates (48 x 24 x 0.4 cm) as front and back panel are used. In between, rubber foam spacers (48 x 2 x 0.4 cm) are sandwiched at the left and right edge of the two panels. The sandwich is taped together along the side and bottom edges by two layers of Micropore tape (Duchefa; 2.5 cm wide). The first layer of tape is started halfway the sandwich, whereas the last layer should be started from the top of the sandwich. This assembly is fixed by application of 2 slide binders (Pavo; A4 15 mm) along the edge at the sides of the panels, leaving the top and bottom exposed. An overview is given in Figure S1. Potting soil is sieved (mesh pore size 2 mm) before filling of the rhizotrons. Next, a funnel is used to gradually pour the sieved soil into the rhizotron till it is filled. Compaction of the soil is achieved by generating a downward force by tapping the rhizotron onto the floor. The soil is compacted until the soil surface reaches 1/2 of the rhizotron height. The rhizotron is then filled up to the top in the same manner and compacted as previously, according to the following heights: 1/3, 3/4, 7/8 and 15/16. During the last addition of soil, a layer of soil is put on top of the rhizotron twice before it is pushed into the gap between the two panels. This process of filling/compacting is being monitored by weighing using a balance with approximately the following weights per step; 110 g (1/2), 160 g (1/3), 210 g (3/4), 230 g (7/8), 240 g (15/16) and 250 g (full). Sealing of the top is achieved by applying one layer of Micropore tape (Duchefa; 2.5 cm wide). The filled rhizotrons are submerged overnight in a nutrient bath (Hyponex, Hydro Agri Specialties) in vertical position. The next day the rhizotrons are taken out of the nutrient solution, placed upright under an angle of 43° and left to drain for 2 hours in order to remove the excess solution. Both sides of the rhizotron are covered with aluminium foil to emulate a below-ground environment for the plant roots. Finally, the rhizotrons are transferred to boxes and kept at a 43° angle for the duration of the experiments.

Data S2 Description of Manual Corrections in Root Architecture Analysis

Two types of manual corrections were made for the root architecture analysis. The first type of correction was for the shape-based approach with plate-grown seedlings in reflective scans. The second type of correction was for the trait-based approach with broken primary roots in contrast scans.

On several occasions the root tips of the seedlings grown on large agar plates would touch the bottom at 18 to 20 dpg. Instead of growing straight down the root tip would bend left or right due to space limitation (Figure S2A). The shape-based approach requires positioning of landmarks on fixed points on the root system, of which the lowest root tip is one (landmark 5). In affected tips, uncorrected landmark 5 (Figure S2B, red dot) would not represent the true root system shape due to the bending initiation (Figure S2B, black dot). A correction was made by first measuring the length of the root from bend initiation to the root tip, and then drawing a straight line (Figure S2C, green line) with corresponding length in the same direction from the final unbent segment. At the end of this line, the corrected landmark 5 (Figure S2C, green dot) was positioned.

For the trait-based analysis the root systems were isolated from their experimental system (plate or rhizotron) to make contrast scans. Due to the fragility of the root system, we could not prevent all roots from breaking. The complete root system (both main part and broken pieces) were included in the contrast scan for total root length analysis with WinRhizo (Figure S3A). Since we only traced secondary roots initiation points, broken secondary roots do not have any consequences for further analysis with SmartRoot. Broken primary roots are problematic and therefore digital repair was needed. Broken primary roots were identified by the lack of a root meristem (Figure S3B). In case the primary root was broken, the contrast scan was screened for the presence of separated roots. If separate roots were present, the the remainder of the primary root was determined by critically assessingthickness and secondary root occupancy (Figure S3C). Stitching of the two primary root segments was only performed if pieces could be matched with absolute certainty. In all other cases, the scan was discarded.
